# Outcome of Percutaneous Intervention in Dysfunctional Loop versus Straight Arteriovenous Grafts in Hemodialysis Patients

**DOI:** 10.5334/jbsr.2146

**Published:** 2020-09-09

**Authors:** Eung Tae Kim, Soon-Young Song, Young Kwon Cho

**Affiliations:** 1 Hanyang University Guri Hospital, KR; 2 Hanyang University Hospital, KR; 3 Kangdong Seong-Sim Hospital, Hallym University College of Medicine, KR

**Keywords:** hemodialysis, arteriovenous fistula, graft, percutaneous transluminal angioplasty

## Abstract

**Objective:**

To compare the patency control of dysfunctioning forearm arteriovenous graft (AVG) using percutaneous transluminal angioplasty (PTA) in patients with loop versus straight grafts.

**Materials and Methods:**

Between January 2012 and March 2017, hemodialysis patients with forearm AVG were treated with PTA at two hospitals. We reviewed technical and clinical success rates of each procedure. Procedure time and patency of the graft were compared for all patients as well as for subgroups of stenosis only and thrombosis using paired-sample t-test and Kaplan-Meier analysis.

**Results:**

Sixty-six patients (mean age, 62.11 ± 11.85 years) underwent PTA. Thirty-eight patients (58%) had loop grafts and 28 (42%) had straight grafts. Among 66 patients, 54 (82%) had thrombosis. Technical success rate was 95.5% (only stenosis: 100%; thrombosis: 94%) and the mean procedure time was 48.00 ± 16.75 minutes in all patients. Although there was a tendency towards shorter procedure time in patients with loop grafts (45.24 ± 20.24 minutes) than those with straight grafts (51.85 ± 22.76 minutes), the difference was not statistically significant (*p = 0.217*), with or without thrombi. There was no statistical significance in primary and assisted primary patency (log rank 0.78, *p = 0.38* in primary patency; log rank 0.88, *p = 0.35* in assisted primary patency).

**Conclusion:**

Our study suggests there is no different patency outcome between straight and loop arteriovenous grafts after PTA.

## Introduction

Hemodialysis plays an important role in patients with chronic kidney disease [1,2]. According to internationally recognized guidelines, in patients who need hemodialysis, it is reasonable to have arteriovenous access such as arteriovenous fistula or arteriovenous graft (AVG). It is further suggested that if patients have enough time, native arteriovenous fistula is preferable to an AVG related to superior patency rate [3]. AVGs are created as either an upper arm straight graft with communication between the brachial artery with axillary or basilic vein, or a forearm loop graft with communication between the brachial artery and antecubital vein in the antecubital fossa. In some cases, forearm straight grafts are developed with communication of the radial artery with the antecubital, basilic, or brachial veins [4,5,6]. The type of AVG is dependent on the patient’s vascular condition and the surgeon’s preference.

In the past, treatment of dysfunctional AVGs required surgical revision [7,8]. With the development of percutaneous transluminal angioplasty (PTA), one-year patency of grafts has been extended to more than 20% [2,9,10,11,12,13]. However, there is a definite distinction in the type of procedure used between the two common types of graft in forearm (loop and straight grafts). To our knowledge, there have been no reports comparing the outcome of percutaneous treatment for two different types of AVG in the forearm.

In this study, we compared the effectiveness of PTA for dysfunctioning forearm AVG between patients undergoing hemodialysis with loop and straight grafts.

## Materials and Methods

### Patients

This retrospective study was approved by institutional review boards, and the requirement for written informed consent was waived. From January 2012 to March 2017, hemodialysis patients with AVG failure treated by PTA were retrieved from the records of two hospitals (Hanyang University Hospital and Hanyang University Guri Hospital). Failed hemodialysis was first diagnosed by physical exam or during hemodialysis. Indications for PTA were elevated venous pressure causing difficulty in hemostasis, abnormal urea recirculation (more than 20%), abnormal physical findings (i.e., pulsatile, abnormal thrill on palpation and high pitched, discontinuous, or systolic bruit on auscultation) and unexplained decrease in dialysis dose. Contraindications to PTA were graft infection or severe aneurysmal dilatation.

All patients were treated by interventional radiology in dedicated angiographic suites. We included the patients with an AVG in the forearm. Patients with AVGs in the upper arm or lower extremities were excluded. In addition, graft revisions performed by doctors other than interventional radiologists were excluded. Demographics, clinical data, and operation data for all patients were collected from patients’ electronic medical records from the hospital database.

### Procedure

All interventional procedures were performed by four trained interventional radiologists with 3 to 20 years of experience in intervention radiology. Before the procedure, careful physical examination was performed in all patients with or without an ultrasound assist, to evaluate the location of stenosis or thrombosis. Pre-procedural antibiotics were not routinely administrated. Local anesthesia was administered at the puncture site using 2% lidocaine (Jeil Pharmacy, Seoul, Korea).

**Figure 1:**
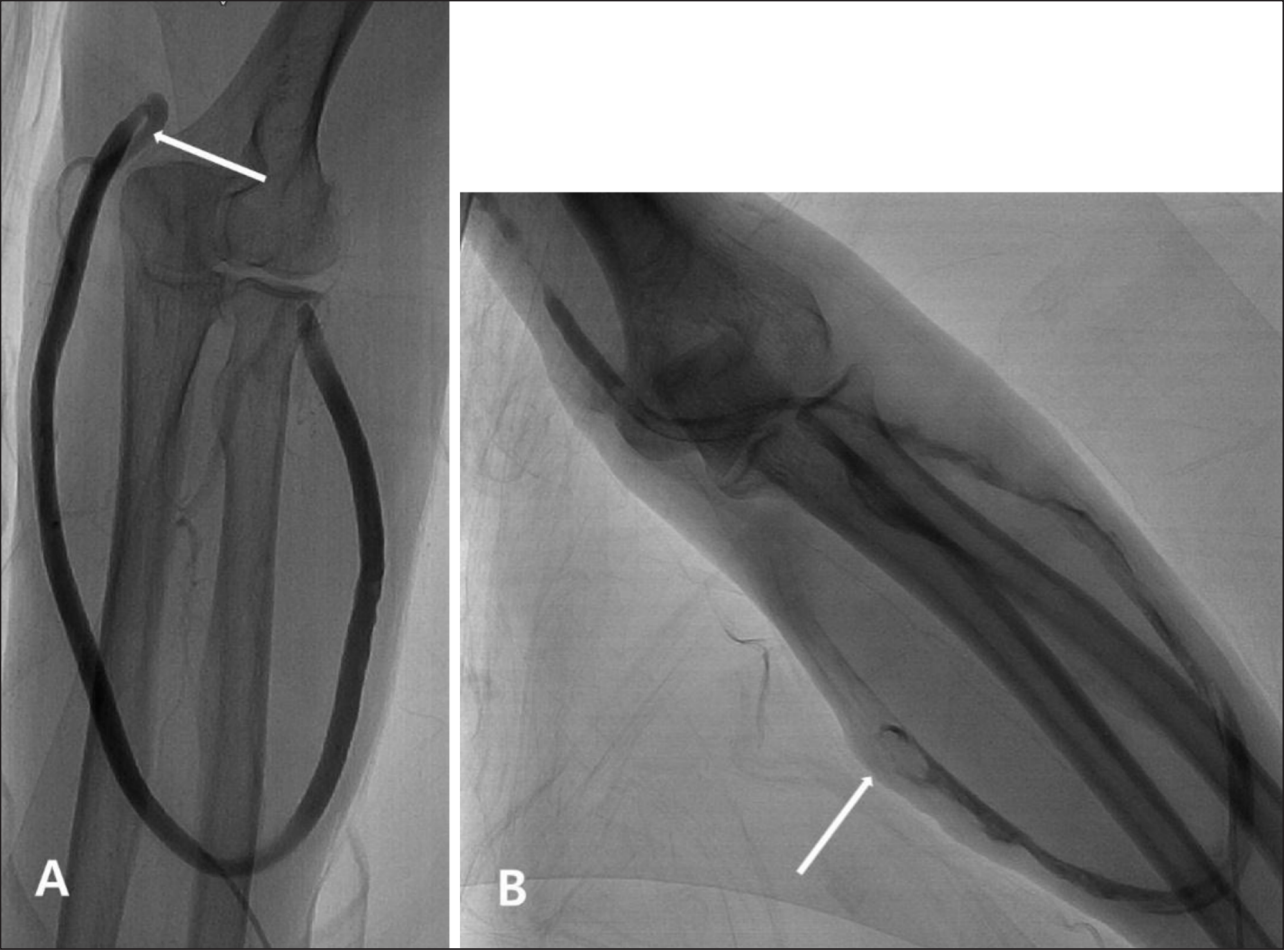
**Percutaneous transluminal angioplasty in patients with loop grafts. (a)** Patient with brachio-cephalic loop graft with focal stenosis (arrow) at arterial anastomosis without thrombosis. Percutaneous balloon angioplasty was performed with one single puncture at apex. The total procedure time was 24 minutes. **(b)** Patient with brachio-cephalic loop graft with multifocal thromboses (arrow) in graft. Aspiration thrombectomy and balloon angioplasty was performed with single puncture at apex. The total procedure time was 33 minutes.

In cases with loop grafts, a single puncture of the graft apex was performed with a micro-puncture set (Semyeong Medical, Seoul, Korea). After accessing the graft, fistulography was performed to evaluate the stenotic lesion and the extent of thrombus. In patients with thrombus, aspiration thrombectomy was first attempted with 7 Fr Hoffman sheath (Cook, Bloomington, IN). If aspiration was not successful, an additional device such as the Trerotola device (Arrow International, Reading, PA) was used to dissolve the thrombosis. After thrombectomy, balloon PTA was carried out with a 6 to 8 mm conventional (Boston Scientific, Marlborough, MA) or high pressure balloon catheter (BD, Franklin Lakes, NJ) in each direction of the arterial and venous sides for one minute. In cases of arterial plug, a Fogarty balloon catheter (Edwards Lifescience, Irvine, CA) was used to remove the plug, or a balloon PTA was used to compress the plug. In all procedures in loop-graft cases, a single puncture was sufficient for the procedure in each direction (Figure 1).

**Figure 2:**
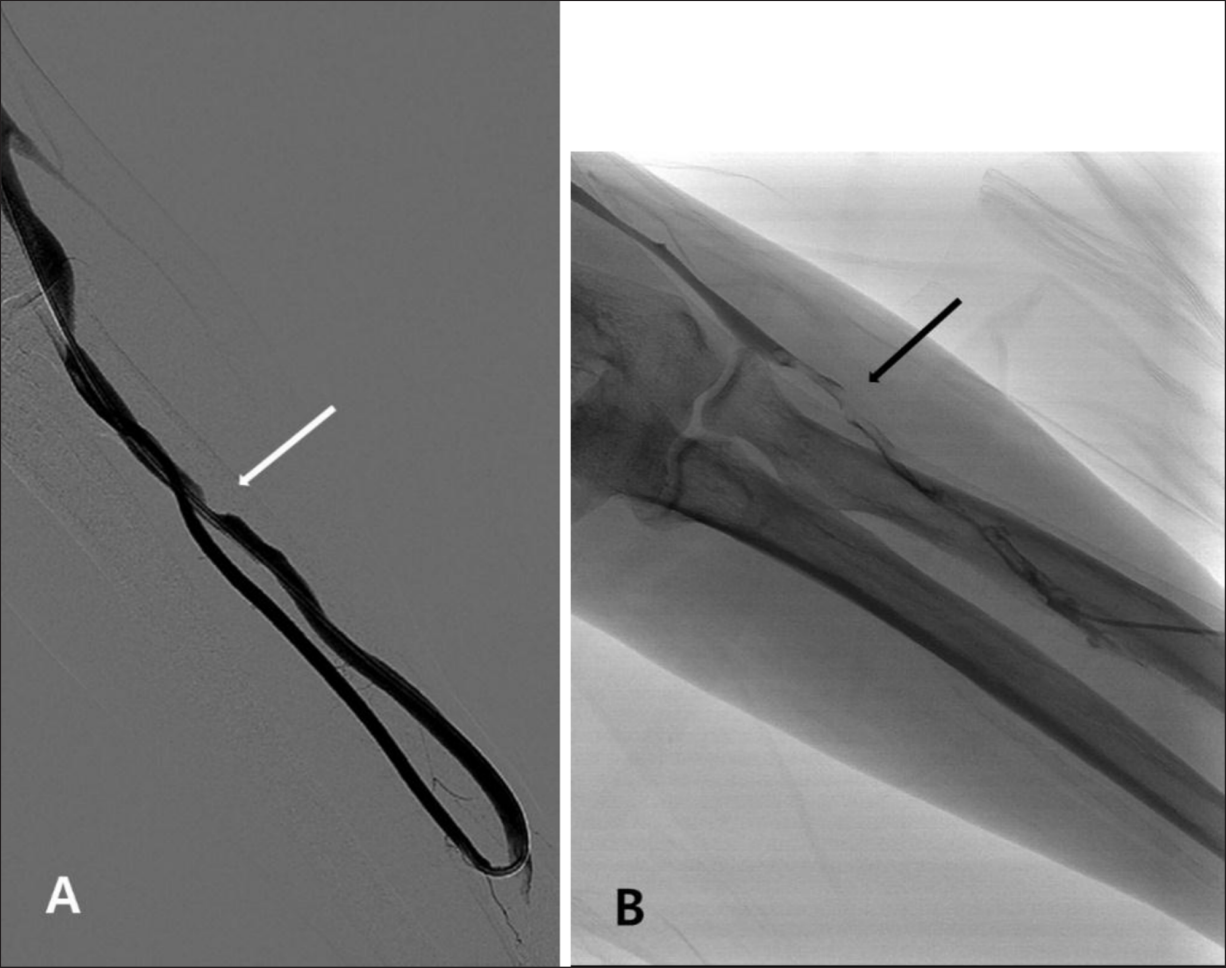
**Percutaneous transluminal angioplasty in patients with loop graft. (a)** Patient with radio-cephalic straight graft with focal stenosis (arrow) at venous anastomosis without thrombosis. Percutaneous balloon angioplasty was performed with one single puncture. The total procedure time was 18 minutes. **(b)** Patient with radio-cephalic straight graft with multifocal thrombosis (arrow) in graft. Additional puncture was done for the opposite side of the direction for aspiration thrombectomy and balloon angioplasty. The total procedure time was 50 minutes.

In straight graft cases, after pre-procedural evaluation, the puncture site was identified. Because of the straight configuration of the graft, changing direction to treat both arterial and venous side was more challenging. In these cases, additional puncture was needed for the opposite direction. The remainder of the procedure was similar to the loop type graft (Figure 2).

### Outcome Analysis

From medical records and the Picture Archival Communication System (PACS), procedure time for each intervention was recorded. With the PACS system, we reviewed technical success rate of the intervention. From medical records, the clinical success rate of hemodialysis after the procedure was recorded. Comparison was performed between loop- and straight graft cases for mean procedure time and success rates.

Evaluation of the patients’ graft status and symptomatic lesion was conducted by two radiologists using the PACS system. Based on this information, patients were divided into two groups, stenosis without thrombosis, and thrombosis with or without stenosis. In each group, comparison was made between the two different types of graft. We recorded the dates of procedure and follow-up procedures to evaluate the primary patency rate and assisted primary patency rate of the graft.

### Statistical Analysis

Statistical analyses were performed using SPSS software, version 21.0. Paired-sample t-test was used to compare the technical success rate and procedure time between the loop graft and straight graft in all patients and each subgroups of stenosis only and thrombosis. Primary and assisted primary patencies of the graft were estimated using Kaplan-Meier technique with life table analysis. Univariate log-rank analysis was used to compare the loop-graft and straight-graft groups. The level of statistical significance was set at p-value < *0.05*.

Technical success was defined as less than 30% residual stenosis of treated lesion in only stenosis patients. In thrombosis patients, technical success was defined as restoration of flow with less than 30% residual stenosis for the associated stenotic segment. Clinical success was defined as successful hemodialysis at least one session after the intervention procedure [14]. We defined procedure time as the time interval between the first venography or scout image and the last venography. Primary patency was defined as patency between the primary intervention and repeated radiologic intervention due to dysfunction in hemodialysis vascular access. Assisted primary patency was defined as patency between primary intervention and until access thrombosis or the time of measurement of patency to maintain the functionality of patent access [14,15]. Surgical interventions for maintenance of patency were excluded.

According to quality improvement guidelines, minor complications are considered when there is no additional therapy required, or only nominal therapy is required, which includes overnight admission for observation only. Major complications are defined when patients required unplanned increased level of care and hospitalization of more than 48 hours [14].

## Results

In total, 66 patients (34 men, 32 women; mean age 62.1 years; range, 20–90 years) underwent interventions for malfunctioning forearm AVG. Of these, 38 (58%) had loop grafts and the remaining 28 (42%) were straight-graft patients. The most common type of AVG was the brachio-cephalic loop graft (n = 12, 18.2%) and radio-cephalic straight graft (n = 12, 18.2%). Graft types are listed in Table 1. Among all patients, 12 had only stenosis without thrombosis and 54 had thrombosis with or without stenosis. The majority of the patients had venous anastomosis site stenosis (n = 59, 89.4%). Arterial obstruction was noted in 20 patients (30.3%). Among 54 patients who had thrombosis, all patients had thrombus in the graft (Table 2). Double puncture was performed in eight patients with straight grafts and no additional puncture was performed in loop-graft cases.

**Table 1:** Types of forearm arteriovenous graft in 66 patients.

	Type of graft	Number	Number of patients with stenosis only

Loop graft	Brachio-antecubital	10	2
	Brachio-basilic	7	1
	Brachio-cephalic	12	3
	Radio-antecubital	1	0
	Radio-basilic	3	1
	Radio-cephalic	5	1
	Total	38	8
Straight graft	Radio-antecubital	5	1
	Radio-basilic	11	1
	Radio-cephalic	12	2
	Total	28	4

**Table 2:** Characteristics of thrombosis and stenotic lesions in graft.

		Thrombosis			Stenosis only		

		Loop	Straight	Total	Loop	Straight	Total

Sites of stenosis	Venous anastomosis	28	22	50	6	3	9
	Draining vein	7	6	13	3	1	4
	Graft	0	0	0	1	0	1
Sites of thrombi	Graft	30 (1)	24 (2)	54 (3)			
	Draining vein	4	2	6			

*Note*: Data in parentheses is number of patients without stenosis.

Technical and clinical success rate in all patients was 95.5% (63/66) and 97.0% (64/66), respectively. Technical success rate of both loop grafts (97.3%) and straight grafts (92.9%) was not different (*p = 0.426*). The mean procedure time was 48.00 ± 16.75 minutes (range: 14–110 minutes) in all patients. Between the two different types of grafts, the mean procedure time in patients with loop grafts (45.24 ± 20.24 minutes) tended to be shorter than in patients with straight grafts (51.85 ± 22.76 minutes), but the difference was not statistically significant (*p = 0.217*). In analysis of the subgroups, there was no statistical significance in technical success rate or mean procedure time between loop- and straight-graft cases (Table 3).

**Table 3:** Technical success rate and mean procedure time of patients.

		Overall	Loop	Straight	*p*-value

Total	Technical success rate	63/66 (95.5%)	37/38 (97.3%)	26/28 (92.9%)	0.426
	Mean procedure time (minutes)	48.00	45.24	51.85	0.217
Stenosis only	Technical success rate	12/12 (100%)	8/8 (100%)	4/4 (100%)	1.000
	Mean procedure time (minutes)	36.00	38.25	32.25	0.610
Combined thrombosis	Technical success rate	51/54 (94%)	29/30 (96.7%)	22/24 (91.7%)	0.220
	Mean procedure time (minutes)	50.67	47.10	55.10	0.170

*Note*: Data in parentheses is percentage.

**Figure 3:**
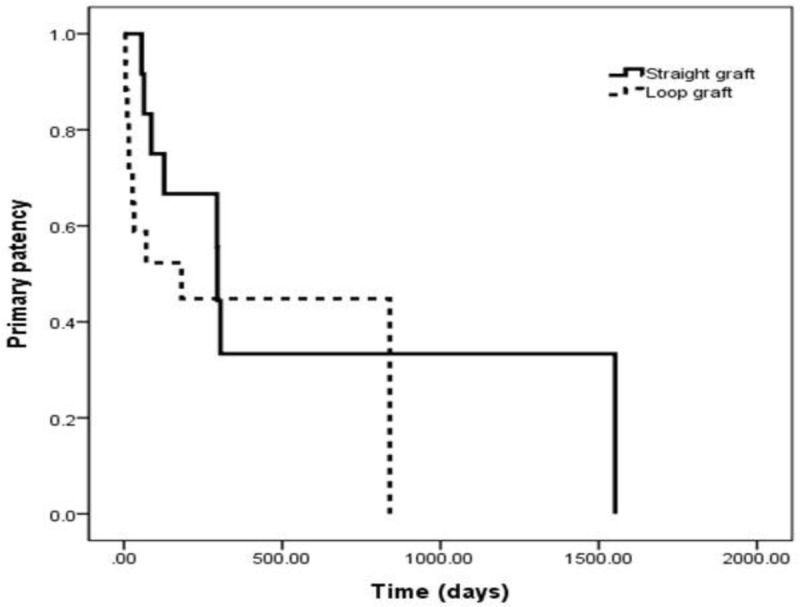
Kaplan-Meier curve showing primary patency of loop and straight grafts.

**Figure 4:**
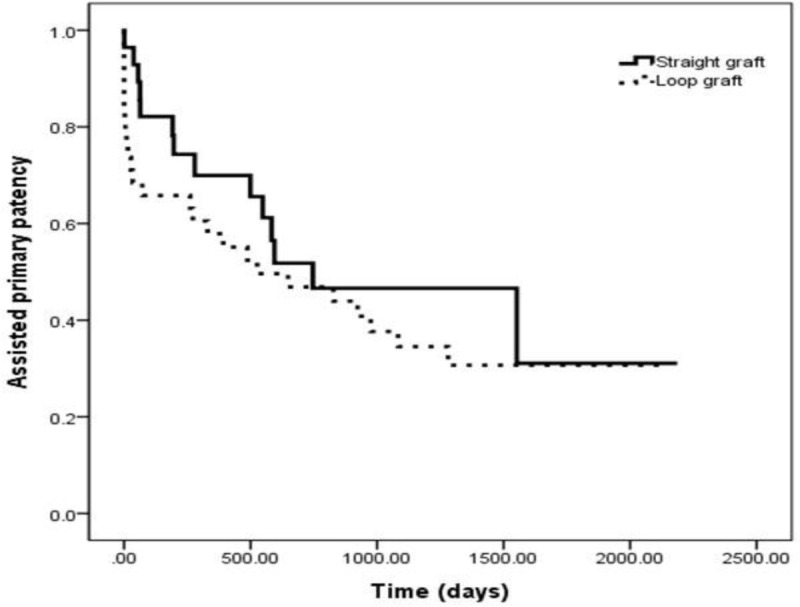
Kaplan-Meier curve showing assisted primary patency of loop and straight grafts.

Primary patency of loop grafts (mean: 193.9 ± 247.7 days) was shorter than that of straight grafts (mean: 304 ± 394.1 days) (Figure 3). Primary patency of straight grafts at 6 and 12 months was 66.7% and 33.3%, respectively, and that of loop grafts at 6 and 12 months was 52.3% and 44.8%, respectively. However, there was no statistical significance (log rank 0.78, *p = 0.38*). The assisted primary patency of loop grafts (mean: 677 ± 652 days) was longer than that of straight grafts (mean: 644 ± 590 days) (Figure 4). Assisted primary patency of straight grafts at 6, 12, 24, and 36 months was 78.2%, 65.6%, 45.6% and 31.1%, respectively, and that of loop grafts was 63.2%, 55.1%, 43.9% and 30.7%, respectively, and there was no statistical significance among the two groups (log rank 0.88, *p = 0.35*).

A major complication occurred in one patient (1/66, 1.5%). During the procedure, the distal tip of the Hoffman sheath fractured and embedded in the graft. However, hemodialysis was completed successfully. Following successful hemodialysis, the patient underwent surgical removal of the fractured tip.

## Discussion

With the rising incidence of end-stage renal disease, management of vascular access is increasingly important in long-term hemodialysis patients [4,12]. However, clinically there are limitations of the hemodialysis graft resulting from progressive stenosis by intimal hyperplasia, leading to thrombosis [2,16]. PTA has been recommended as a better option for treatment, and many published articles have shown improvements in technical success and patency rates [2,9,14]. The current study shows a technical success rate (95.5% [63/66]) in the upper range of those previously reported (79 to 97.8%) [2,17]. The present study showed that most of the graft stenosis occurred in venous anastomosis site, also consistent with previous studies [16,18].

There are several types of AVG used depending upon a patient’s vascular condition. The most commonly used AVG are loop and straight grafts [4,5,6]. To our knowledge there has been no published report comparing the procedure time when evaluating the feasibility of the procedure. Because of the AVG conduit itself, it is expected that there should be differences in procedure type and technique. In loop grafts, most of the procedure could be done using single puncture at the apex. However, in straight-graft cases, there can be some difficulties during the procedure. First, in cases that require changing direction between the arterial and venous sides, occlusion of the lumen could occur by folding of the graft. Second, because of the short length of straight grafts, using a single puncture tends to reduce the distance from anastomosis in one direction. In addition, the small diameter and differences in the distal arterial anastomosis site, along with the acute angle of the anastomosis site, add to the challenges of handling devices such as high pressure balloon or Fogarty balloon catheters. Therefore, many cases require an additional puncture.

In our study, the procedure time in loop-graft cases was shorter than in straight-graft cases, although the difference was not statistically significant. The trend towards a shorter procedure time in loop-graft cases could be explained by previously described procedural differences. In contrast, in the stenosis group, the procedure time was shorter in straight type grafts. This may be because the pre-procedure examination of the graft for the exact location of the stenosis was easier, allowing the treatment of only the problematic stenotic lesion without changing direction. In these situations, the procedure time could be shorter than in cases with combined thrombosis which may need additional treatment.

As reported in previous studies, the one-year patency rate of loop grafts was higher than that of straight grafts (44.8% vs. 33.3%). According to previous studies, the one-year patency rates of the loop graft were reported as 26–31% and those of the straight graft were 17% [2,12]. The authors speculated that loss of energy within the graft is associated with less turbulence, reducing the rate of intimal hyperplasia in the graft [2]. However, our results showed that one-year assisted primary patency of the straight graft (65.6%) was higher than that of the loop graft (55.1%).

According to a previous randomized trial from Katsanos et al., a drug-coated balloon angioplasty improves patency of venous stenosis of dysfunctioning arteriovenous access [19]. Also, randomized trial result from Haskal et al. shows stent-graft use in venous anastomotic stenosis revision at hemodialysis graft provide long-term and patency than standard balloon angioplasty [20]. However, current guidelines state inadequate evidence to recommend drug-coated balloon [3]. Proper application of these devices could improve patency of the arteriovenous access of hemodialysis.

There are several limitations in this study. First, this study is retrospective in nature and is subject to selection bias. All data such as minor complications might not required and properly recorded in all patients. And there may have intervention and outcome measurement error to occur. Additionally, because of lack of random allocation, there were differences in the perspectives of many factors influencing the outcome. Further study with randomized clinical trials is required. Secondly, several operators performed the procedures with anywhere from 3 to 20 years of experience in the interventional field. There was likely to be a difference in proficiency with the procedure based on experience. Third, the number of the patients was small, possibly introducing statistical error. Finally, since this study included only forearm grafts, further study is needed to generalize the results with graft conduits.

## Conclusion

Despite the anticipated differences in technical difficulties, our study did not show significant dissimilarity in success rate, procedure time, and patency rate of PCA between the two types of AVG. Further and larger studies are needed to confirm that there are no differences in outcome of PTA in dysfuctioning straight and loop grafts.

## Abbreviation

PTA = percutaneous transluminal angioplasty

AVG = arteriovenous graft
